# Human Umbilical Cord MSCs as New Cell Sources for Promoting Periodontal Regeneration in Inflammatory Periodontal Defect: Errautm

**DOI:** 10.7150/thno.74942

**Published:** 2022-07-08

**Authors:** Fengqing Shang, Shiyu Liu, Leiguo Ming, Rong Tian, Fang Jin, Yin Ding, Yongjie Zhang, Hongmei Zhang, Zhihong Deng, Yan Jin

**Affiliations:** 1 State Key Laboratory of Military Stomatology & National Clinical Research Center for Oral Diseases & Shaanxi Key Laboratory of Oral Diseases, Center for Tissue Engineering, Fourth Military Medical University, Xi'an, Shaanxi 710032, China;; 2 Research and Development Centre for Tissue Engineering, Fourth Military Medical University, Xi'an, Shaanxi, China;; 3 Department of Stomatology, the 306th Hospital of PLA, Beijing 100101, China;; 4 State Key Laboratory of Military Stomatology &National Clinical Research Center for Oral Diseases & Shaanxi Clinical Research Center for Oral Diseases, Department of Orthodontics, School of Stomatology, The Fourth Military Medical University, Xi'an, Shaanxi, 710032, China;; 5 Department of Burns and Plastic surgery, Tangdu Hospital, Fourth Military University, Xi'an, Shaanxi, 710038, China;; 6 Department of Otolaryngology, Xijing Hospital, Fourth Military Medical University, Xi'an, Shaanxi, 710032, China.

The authors apologize that the original version of the above article contains errors that need to be corrected. Incorrect images for Figure 4F (bottom) and 4G (bottom) were used in figure assembly. The authors apologize for any inconvenience these errors may have caused. Luckily the correction does not affect the conclusions of the above paper. The corrected Figure 4F and 4G appear below.

## Figures and Tables

**Figure 4:**
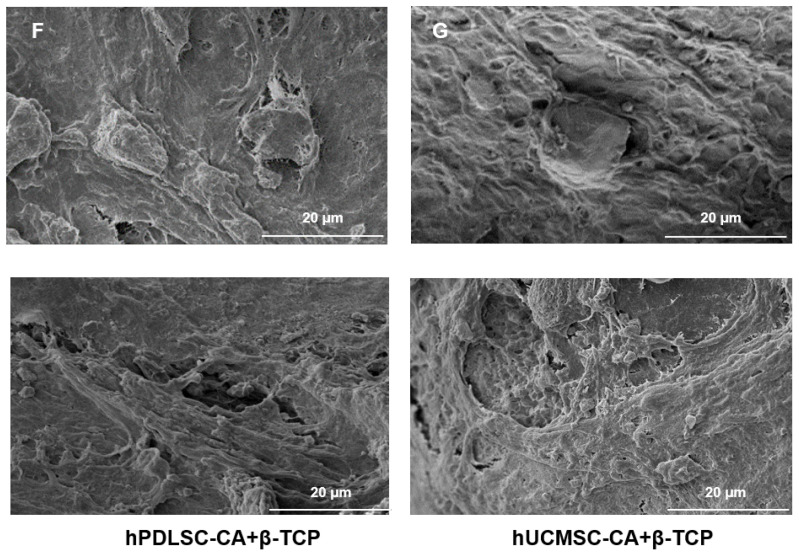
Corrected figure for original Figure 4F and 4G.

